# Health Tract, No. 147

**Published:** 1863-07

**Authors:** 


### HEALTH TRACT, No. 147.

### DIETING

Is usually considered to mean the same thing as a kind of starvation. The idea which the educated physician attaches to the term is a judicious regulation of the quantity and quality of the food, according to the circumstances of each case. A healthy man may diet himself in order to keep well; an invalid may diet himself with a view to the recovery of his health; yet the things eaten by the two will widely differ in their nature, bulk, and mode of preparation. A vast multitude are suffering hourly by the horrors of dyspepsia; no two are precisely alike in all points, since there is an endless variety of combinations as to age, sex, occupation, air, exercise, mode of eating, sleeping, constitution, temperament, etc. Yet dyspepsia is always brought on by over and irregular eating; it could be banished from the world in a generation, if the children were educated to eat moderately, regularly, and slowly; the parents who do this will do their offspring a higher good than by leaving them large fortunes, which, in three cases out of four, foster idleness, gluttony, and every evil thing. As the rich can get any thing to eat or drink when they want it, they, with indulged children, bring on dyspepsia by eating irregularly and without an appetite. The poor—those who have to work for a living—induce the horrible disease by eating too rapidly and at unseasonable hours; mainly by eating heartily at supper, and going to bed within an hour or two afterward. In the heyday of youth and manly vigor there may not for a while be noticed any special ill effect from such a practice—in truth, it is at first inappreciable, but it is cumulative, and impossible not to manifest itself in due time. Infinite Benevolence forgives a moral delinquency; but omnipotent as he is, and loving toward all, it is not in the nature of his government of created things to work a miracle, to suspend a natural law, in order to shield one of his creatures from the legitimate effects of a violence offered the physical system by excesses in eating, drinking, or exercise.

Perhaps hearty suppers make more dyspeptics than any or all other causes combined. If dinner is at noon, nothing should be taken for supper but a single cup of weak tea, or other hot drink, and a piece of stale bread and butter. After forty years of age, those who live in-doors, sedentary persons—that is, all who do not work with their hands as laborers—would do better not to take any supper at all. Half-the time the sedentary, who eat at noon, do not feel hungry at supper; especially if they see nothing on the table but bread, and butter, and tea. But nature is goaded to act against her instincts in almost every family in the nation by "relishes" being placed on the supper-table, in the shape of chipped beef, salt fish, cake, preserves, or other kinds of sweetmeat, and before the person is aware, a hearty meal has been taken, resulting in present uncomfortableness, in disturbed sleep, in a weary waking in the morning, bad taste in the mouth, and little or no appetite for breakfast, all of which can be avoided by beginning early to eat habitually, according to the suggestions above made.

